# Supplementary material for: Characteristics of individuals who received a complete, 2-dose mpox vaccine regimen as part of the public health response to the mpox epidemic in Ontario, Canada
Source: PLOS Glob Public Health. 2025 Nov 26;5(11):e0005452. doi: 10.1371/journal.pgph.0005452 (PMC12654912; doi:10.1371/journal.pgph.0005452)
Supplement: S1 Text — (DOCX) [file pgph.0005452.s001.docx]

**S1 Text**

## **Linked data repositories**

Ontario has a single-payer health system that provides universal access to physician and hospital services and laboratory testing for all eligible Ontario residents. For this study, we used population-based databases that capture relevant information within this single-payer system.

MVA-BN was administered as pre-exposure prophylaxis to eligible high-risk groups by local public health units at mass immunization clinics in coordination with community-based organizations, with a smaller number administered via physician clinics. The mass immunization clinics were implemented in drop-in settings, including community-based organizations, with a low-barrier approach to accelerate uptake and maximize access. The low-barrier approach meant that confirmation of eligibility criteria was not required, and any individual who came to the mpox-specific mass immunization clinic could receive the vaccine.

The provincial Digital Health Immunization Repository (DHIR) contains individual-level vaccination data (e.g., product, dose, site, date) entered at the time of administration. Data on post-exposure prophylaxis doses administered by primary care providers were submitted to local public health units for entry into DHIR retrospectively.

All mpox specimens collected by health care providers were submitted to Public Health Ontario laboratories for testing. Laboratory results and demographic and clinical data from the test requisition were obtained from LabWare, Public Health Ontario’s laboratory information system.

Data on syphilis testing were obtained from LabWare and the Ontario Laboratories Information System (OLIS), a provincial repository of laboratory testing results from hospital laboratories, community-based commercial laboratories, and public health laboratories. Data on prior diagnoses of bacterial sexually transmitted infections (gonorrhea, chlamydia, and syphilis) were obtained from the integrated Public Health Information System (iPHIS), an information system for the reporting and surveillance of Diseases of Public Health Significance.

History of HIV diagnosis was ascertained from the ICES-derived HIV Cohort, which uses a validated algorithm based on physician office visits with HIV as the diagnostic code to identify individuals living with HIV.^1^ Additionally, HIV pre-exposure prophylaxis prescription data were obtained from the Ontario Drug Benefit (ODB) database, which contains information on all publicly-funded prescriptions for: adults aged ≥65 years; recipients of professional home services and social assistance; children and young adults aged ≤24 years and not covered by a private insurance plan; and recipients of the Trillium Drug program, which helps Ontarians who have high prescription drug costs relative to household income.^2,3^

Information on COVID-19 vaccination was obtained from Ontario’s centralized province-wide COVID-19 vaccine registry, COVaxON. Data on influenza vaccines and other publicly funded vaccines received in physician offices were extracted from the Ontario Health Insurance Plan (OHIP) physician billing claims database, while information on influenza vaccines received in pharmacies was extracted from the ODB database. Data on number of physician office visits and whether individuals had a primary care physician were also obtained from the OHIP physician billing claims database. Data on immunocompromised status was obtained from CCI procedure codes and OHIP feecodes, ODB to identify immunosuppressive medications, the OCR for cancer diagnoses, and Discharge Abstract Database (DAD), Same Day Surgery (SDS), OHIP, and National Ambulatory Care Reporting System (NACRS) for other healthcare encounters that suggested individuals had disorders of the immune system.

We obtained age, sex, postal code, and neighborhood-level income and visible minority quintile from the Ontario Registered Persons Database (RPDB), a population registry with demographic information for all Ontarians with provincial health insurance. We determined the geographic region (public health unit, which we then collapsed into a smaller number of regions) using the postal code and Statistics Canada Postal Code Conversion File plus (version 7B). We determined immigration status using the Immigration, Refugees and Citizenship Canada (IRCC) Permanent Residents Database, which includes administrative information related to temporary and permanent residents in Canada.

De-identified data extracted from repositories held at Public Health Ontario (Panorama, Labware, iPHIS) were securely shared with ICES, a not-for-profit research institution, and linked to health administrative databases held at ICES using unique encoded identifiers. Linked data were analyzed at ICES.

For the continuous variables in Table 1 (age, days between first and second dose, rate of syphilis tests, and rate of bacterial STIs), visual inspection of the distribution of this continuous variable and tests of normality (Kolmogorov-Smirnov, Cramer-von Mises and Anderson-Darling) demonstrated a skewed distribution. Thus, the median and inter-quartile range was reported, and the Kruskal-Wallis test used for comparison across groups.

# **References**

1. Antoniou T, Zagorski B, Loutfy MR, Strike C, Glazier RH. Validation of case-finding algorithms derived from administrative data for identifying adults living with human immunodeficiency virus infection. PLOS One 2011;6:e21748.

2. Ontario Ministry of Health. Ontario Drug Benefit (ODB) Database. Ontario: Queen's Printer for Ontario, 2017 (<https://data.ontario.ca/dataset/ontario-drug-benefit-odb-database>).

3. Ontario Ministry of Health. Get help with high prescription drug costs. Ontario: Queen's Printer for Ontario, 2016 (<https://www.ontario.ca/page/get-help-high-prescription-drug-costs>).
