# Supplementary material for: Characteristics of individuals who received a complete, 2-dose mpox vaccine regimen as part of the public health response to the mpox epidemic in Ontario, Canada
Source: PLOS Glob Public Health. 2025 Nov 26;5(11):e0005452. doi: 10.1371/journal.pgph.0005452 (PMC12654912; doi:10.1371/journal.pgph.0005452)
Supplement: S1 Fig — (DOCX) [file pgph.0005452.s002.docx]

**S1 Fig**


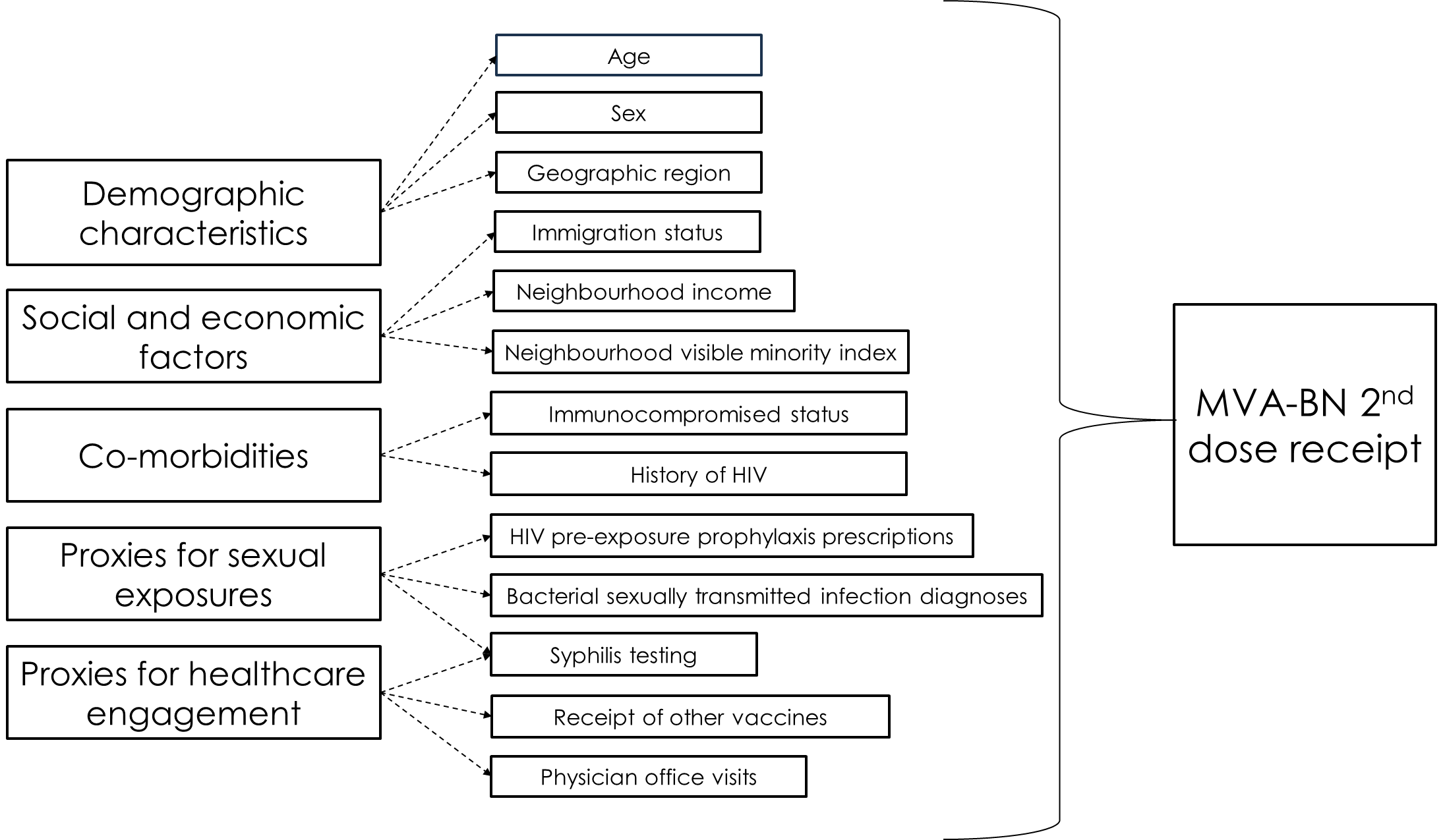


**S1 Fig.** Conceptual framework for associations explored between characteristics and MVA-BN dose 2 receipt.
