# Supplementary material for: Characteristics of individuals who received a complete, 2-dose mpox vaccine regimen as part of the public health response to the mpox epidemic in Ontario, Canada
Source: PLOS Glob Public Health. 2025 Nov 26;5(11):e0005452. doi: 10.1371/journal.pgph.0005452 (PMC12654912; doi:10.1371/journal.pgph.0005452)
Supplement: S2 Fig — (DOCX) [file pgph.0005452.s003.docx]

**S2 Fig**


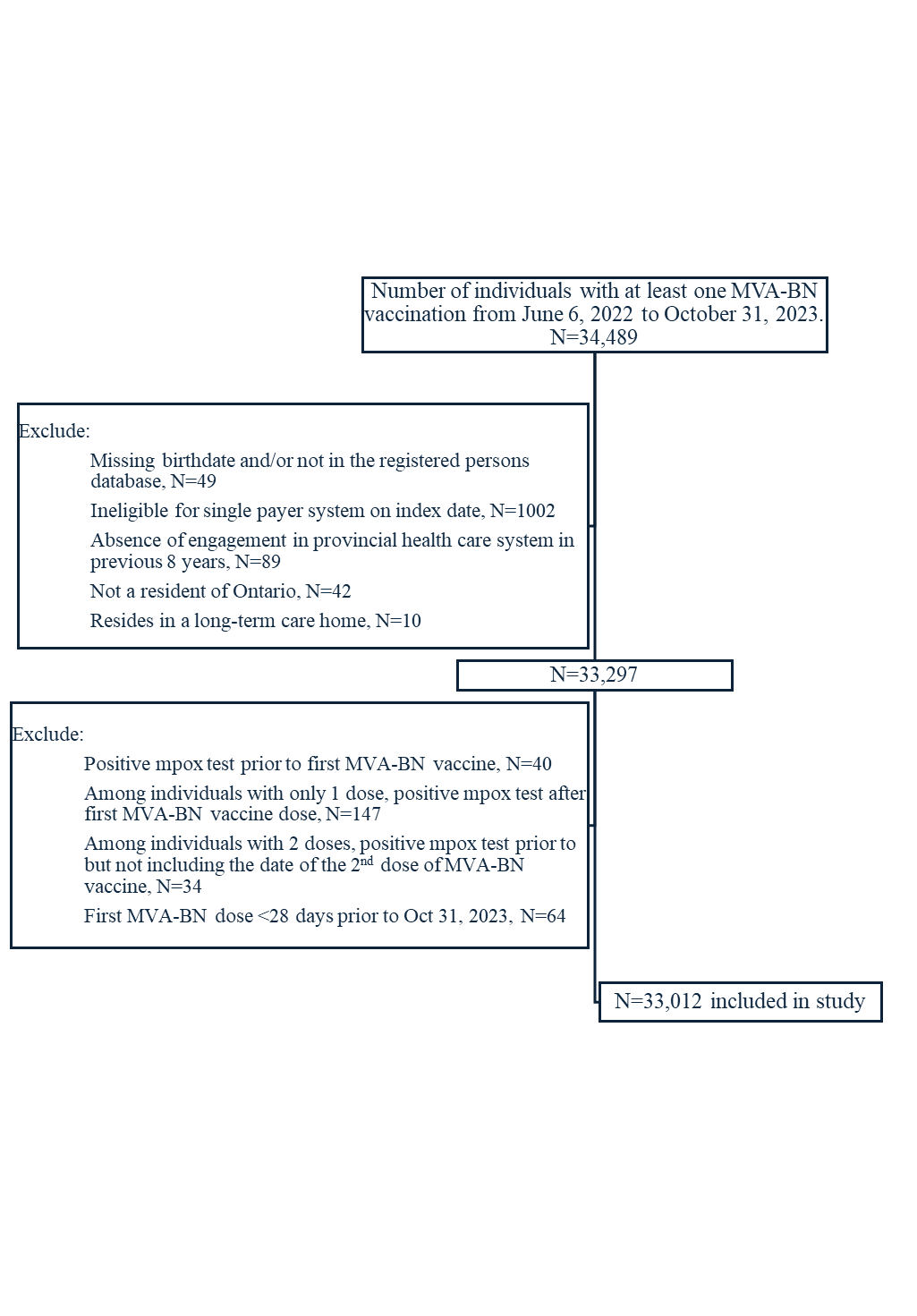


**S2 Fig. Flow chart of population included in study.** The first set of exclusions are based on removing individuals without demographic information, who do not reside in the province (known, or implied based on absence of engagement with the healthcare system) or who reside in long-term care homes who would not have been eligible for MVA-BN unless as post-exposure prophylaxis. The second set of exclusions were to remove individuals with an mpox infection (prior to first dose or after the first dose) and thus, would not have been offered a 2^nd^ dose of MVA-BN based on current vaccine recommendations.
