# Supplementary material for: Characteristics of individuals who received a complete, 2-dose mpox vaccine regimen as part of the public health response to the mpox epidemic in Ontario, Canada
Source: PLOS Glob Public Health. 2025 Nov 26;5(11):e0005452. doi: 10.1371/journal.pgph.0005452 (PMC12654912; doi:10.1371/journal.pgph.0005452)
Supplement: S1 Table — (DOCX) [file pgph.0005452.s005.docx]

# **S1 Table.** Definitions of variables used.

| **Variable** | **Definition** |
| --- | --- |
| Laboratory-confirmed mpox infection (used for exclusion criteria) | First positive or intermediate test result on real time polymerase-chain-reaction (PCR) testing for orthopox/mpox on samples collected from any body site. |
| Reason for immunization | Immunization provided for pre-exposure prophylaxis or post-exposure prophylaxis. |
| Number of days between 1^st^ and 2^nd^ dose of MVA-BN | Days between a patient’s first MVA-BN dose and second MVA-BN dose. |
| First dose before or on/after September 30, 2023 | Indicator of whether a patient had their first MVA-BN dose prior to September 30, 2023 or on/after September 30, 2023. |
| Age-group | Age at dose 1 was determined from the Registered Persons Database, and categorized into the following groups: 0-17 years, 18-24 years, 25-29 years, 30-39 years, 40-49 years, 50-59 years, and ≥60 years. |
| Geographic region (public health unit region) | Ascertained from Public Health Unit (PHU) information using postal code of residence as recorded in the Registered Persons Database and Statistics Canada Postal Code Conversion File Plus (version 7B). Regions were defined as follows:  Toronto: PHU 95 (City of Toronto Health Unit)  Peel/York/Durham/Halton: PHU 53 (Peel Regional Health Unit), 70 (York Regional Health Unit), 30 (Durham Regional Health Unit), 36 (Halton Regional Health Unit)  Hamilton/Niagara/London/Windsor: 37 (City of Hamilton Health Unit), 46 (Niagara Regional Area Health Unit), 44 (Middlesex-London Health Unit), 68 (Windsor-Essex County Health Unit)  Ottawa: PHU 51 (City of Ottawa Health Unit)  Rest of Ontario: PHU 35 (Haliburton, Kawartha, Pine Ridge District Health Unit), 55 (Peterborough County—City Health Unit), 60 (Simcoe Muskoka District Health Unit), 27 (Brant County Health Unit), 34 (Haldimand-Norfolk Health Unit), 36 (Halton Regional Health Unit), 65 (Waterloo Health Unit), 66 (Wellington-Dufferin-Guelph Health Unit), 38 (Hastings and Prince Edward Counties Health Unit), 41 (Kingston, Frontenac and Lennox and Addington Health Unit), 43 (Leeds, Grenville and Lanark District Health Unit), 57 (Renfrew County and District Health Unit), 58 (The Eastern Ontario Health Unit), 26 (The District of Algoma Health Unit), 47 (North Bay Parry Sound District Health Unit), 49 (Northwestern Health Unit), 56 (Porcupine Health Unit), 61 (Sudbury and District Health Unit), 62 (Thunder Bay District Health Unit), 63 (Timiskaming Health Unit), 31 (Elgin-St. Thomas), 33 (Grey Bruce Health Unit), 39 (Huron County Health Unit), 40 (Chatham-Kent Health Unit), 42 (Lambton Health Unit), 54 (Perth District Health Unit), 75 (Southwestern Health Unit) |
| Syphilis testing | One or more syphilis serological screening tests positive in OLIS or LabWare categorized as yes/no or number of tests as 0, 1, 2, 3, and ≥4. Monthly rate of syphilis testing after dose 1 computed for each individual up until they either received their 2^nd^ dose, end of study period (October 31, 2023), or death. |
| Number of bacterial STIs | One or more bacterial STI (gonorrhea, chlamydia, or syphilis) diagnosis from iPHIS categorized as 0, 1, 2, 3, and ≥4. |
| HIV status | An ICES-specific HIV database was used to identify patients with HIV, based on 3 physician claims in 3 years with OHIP diagnostic codes: 042, 043, or 044.^1^ |
| HIV pre-exposure prophylaxis | HIV pre-exposure prophylaxis defined as a dispensation of tenofovir/emtricitabine recorded in the Ontario Drug Benefit (ODB) database, and excluding those prescribed other antiretrovirals within three months of the tenofovir/emtricitabine prescription.^4^ |
| History of receipt of non-MVA-BN vaccines in past one year | COVID-19 vaccines: The date of vaccine receipt from COVaXON.  Influenza vaccines:  Received in physician offices - OHIP feecodes: G590, G591, G592, Q130, Q590, Q690, Q691  Received in pharmacy - ODB billing with any of the following Drug Identification Numbers (DINs): 02420643, 02420783, 02432730, 02473283, 02445646, 02494248, 09857645, 09857646  Other vaccines received in physician offices:  OHIP feecodes: G840, G841, G842, G843, G844, G845, G846, G847, G848, G538, G539 |
| Neighborhood-level income quintiles | Household income quintile calculated at the disseminated area (DA) level was used for the neighborhood-level income.  A dissemination area (DA) is the smallest standard geographic area for which all census data are disseminated. A DA generally comprises approximately 400-700 people, but in densely populated cities may contain several thousand people. DAs cover all the territory of Canada.^3^ We assigned subjects to a DA using postal code, as recorded in the Registered Persons Database.  Calculated at the DA level using 2016 Census data by multiplying the median income (before-tax) by the number of households and dividing by the sum of single-person equivalent to obtain income per single person equivalent.^4^ For DAs where median income was unavailable, neighboring DAs were used to estimate income per single person equivalent. DA-based income quintiles were constructed separately for each census metropolitan area or census agglomeration (one or more adjacent municipalities integrated via commuting flows). DAs within each such area were ranked from the lowest average income per single-person equivalent to the highest, and DAs were assigned to five groups, such that each group contained approximately one-fifth the total in-scope population of each area. |
| Neighbourhood level visible minority quintiles | Those who self-identify as a visible minority. Calculated at the DA level using 2016 Census data. Ranked from lowest to highest and assigned to five groups, |
| Immigration status | Immigration application records for people who initially applied to land in Ontario. The data contains permanent residents' demographic information such as country of citizenship, level of education, mother tongue, and landing date. Categorized as: Refugees, immigration < 5 years, immigration 5-10 years, immigration >10 years, born in Canada or immigrated before 1985 (long-term residents). |
| Has a primary care physician | Whether patient is registered to a physician via the Client Agency Program Enrolment (CAPE) on index date. |
| Number of physician office visits | Any outpatient (office, home, or phone) OHIP visit/consult submitted by any physician via OHIP billing codes. |
| Moderately or severely immunocompromised | Includes Dialysis, Hematopoetic stem cell transplant recipient,  Solid organ transplant recipient, Receiving active treatment for solid tumour or hematologic malignancy, and/or Primary immunodeficiency. |

# **References**

1. Antoniou T, Zagorski B, Loutfy MR, Strike C, Glazier RH. Validation of case-finding algorithms derived from administrative data for identifying adults living with human immunodeficiency virus infection. PLOS One 2011;6:e21748.

2. Ontario Ministry of Health. Ontario Drug Benefit (ODB) Database. Ontario: Queen's Printer for Ontario, 2017 (<https://data.ontario.ca/dataset/ontario-drug-benefit-odb-database>).

3. Ontario Ministry of Health. Get help with high prescription drug costs. Ontario: Queen's Printer for Ontario, 2016 (<https://www.ontario.ca/page/get-help-high-prescription-drug-costs>).

4. Tan DH, Dashwood TM, Wilton J, Kroch A, Gomes T, Martins D. Trends in HIV pre-exposure prophylaxis uptake in Ontario, Canada, and impact of policy changes: a population-based analysis of projected pharmacy data (2015–2018). Canadian Journal of Public Health 2021;112:89-96.
