# Supplementary material for: Characteristics of individuals who received a complete, 2-dose mpox vaccine regimen as part of the public health response to the mpox epidemic in Ontario, Canada
Source: PLOS Glob Public Health. 2025 Nov 26;5(11):e0005452. doi: 10.1371/journal.pgph.0005452 (PMC12654912; doi:10.1371/journal.pgph.0005452)
Supplement: S2 Table — (DOCX) [file pgph.0005452.s006.docx]

**S2 Table.** Descriptive characteristics of individuals who received at least 1 dose of the MVA-BN vaccine in Ontario, Canada, between June 6, 2022, to October 31, 2023, stratified by those who received dose 2 versus those who received only dose 1, and presented with row percentages.

| **Characteristics** | **At least 1 dose, N** | **1 dose only,**  **N(row %)** | **2 doses,**  **N(row %)** |
| --- | --- | --- | --- |
|  | **N=33,012** | **N=20,392** | **N=12,620** |
| First dose before or on/after September 30, 2023 |  |  |  |
| Before September 30 | 29,650 | 18,565 (62.6%) | 11,085 (37.4%) |
|  |  |  |  |
| On/After September 30 | 3,362 | 1,827 (54.3%) | 1,535 (45.7%) |
|  |  |  |  |
| Age group (years) at dose 1 |  |  |  |
| 0-17 | 34 | 29 - 33^a^ | 1 - 5 ^a^ |
| 18-24 | 2,290 | 1766 - 1770 ^a^ | 520 - 524 ^a^ |
| 25-29 | 4,519 | 3,174 (70.2%) | 1,345 (29.8%) |
| 30-39 | 10,754 | 6,633 (61.7%) | 4,121 (38.3%) |
| 40-49 | 6,078 | 3,341 (55.0%) | 2,737 (45.0%) |
| 50-59 | 5,398 | 3,105 (57.5%) | 2,293 (42.5%) |
| ≥60 | 3,939 | 2,340 (59.4%) | 1,599 (40.6%) |
| Sex |  |  |  |
| Female | 2,089 | 1,661 (79.5%) | 428 (20.5%) |
| Male | 30,923 | 18,731 (60.6%) | 12,192 (39.4%) |
| Reason for immunization |  |  |  |
| Post-exposure | 218 | 192 (88.1%) | 26 (11.9%) |
| Pre-exposure | 32,794 | 20,200 (61.6%) | 12,594 (38.4%) |
| Geographic region |  |  |  |
| Toronto | 20,420 | 12,730 (62.3%) | 7,690 (37.7%) |
| Ottawa | 3,689 | 1,927 (52.2%) | 1,762 (47.8%) |
| Peel, York, Durham, Halton | 3,467 | 2,519 (72.7%) | 948 (27.3%) |
| Hamilton, Niagara, London, Windsor | 2,107 | 1,393 (66.1%) | 714 (33.9%) |
| Rest of Ontario | 3,329 | 1,823 (54.8%) | 1,506 (45.2%) |
| Neighbourhood income quintile |  |  |  |
| Missing | 103 | 68 (66.0%) | 35 (34.0%) |
| 1 (lowest) | 7,940 | 4,999 (63.0%) | 2,941 (37.0%) |
| 2 | 7,363 | 4,501 (61.1%) | 2,862 (38.9%) |
| 3 | 6,209 | 3,767 (60.7%) | 2,442 (39.3%) |
| 4 | 5,392 | 3,368 (62.5%) | 2,024 (37.5%) |
| 5 (highest) | 6,005 | 3,689 (61.4%) | 2,316 (38.6%) |
| Neighbourhood visible minorities quintile |  |  |  |
| Missing | 2,195 | 1,378 (62.8%) | 817 (37.2%) |
| 1 (lowest) | 1,226 | 690 (56.3%) | 536 (43.7%) |
| 2 | 2,170 | 1,276 (58.8%) | 894 (41.2%) |
| 3 | 5,576 | 3,372 (60.5%) | 2,204 (39.5%) |
| 4 | 12,618 | 7,750 (61.4%) | 4,868 (38.6%) |
| 5 (highest) | 9,227 | 5,926 (64.2%) | 3,301 (35.8%) |
| Recent immigration |  |  |  |
| Refugees | 1,097 | 562 (62.8%) | 333 (37.2%) |
| <5 years ago | 1,988 | 1,235 (62.1%) | 753 (37.9%) |
| 5-10 years ago | 895 | 2,322 (63.4%) | 1,340 (36.6%) |
| >10 years ago (but after1985) | 3,662 | 15,491 (61.1%) | 9,879 (38.9%) |
| Born in Canada or immigrated before 1985 | 25,370 | 782 (71.3%) | 315 (28.7%) |
| Received any vaccine before dose 1, in past year (COVID-19, influenza, or other) |  |  |  |
| No | 3,362 | 2,216 (77.1%) | 657 (22.9%) |
| Yes | 29,650 | 18,176 (60.3%) | 11,963 (39.7%) |
| Syphilis screening tests before dose 1, past 1 year |  |  |  |
| No | 17,231 | 11,427 (66.3%) | 5,804 (33.7%) |
| Yes | 15,781 | 8,965 (56.8%) | 6,816 (43.2%) |
| Number of syphilis screening tests before dose 1, past 1 year |  |  |  |
| 0 | 17,231 | 11,427 (66.3%) | 5,804 (33.7%) |
| 1 | 5,986 | 3,640 (60.8%) | 2,346 (39.2%) |
| 2 | 3,734 | 2,133 (57.1%) | 1,601 (42.9%) |
| 3 | 2,743 | 1,512 (55.1%) | 1,231 (44.9%) |
| ≥4 | 3,318 | 1,680 (50.6%) | 1,638 (49.4%) |
| Number of syphilis screening tests >3 months^b^ after dose 1 |  |  |  |
| 0 | 15,584 | 10,715 (68.8%) | 4,869 (31.2%) |
| 1 | 5,508 | 3,490 (63.4%) | 2,018 (36.6%) |
| 2 | 3,643 | 2,112 (58.0%) | 1,531 (42.0%) |
| 3 | 3,056 | 1,696 (55.5%) | 1,360 (44.5%) |
| ≥4 | 5,221 | 2,379 (45.6%) | 2,842 (54.4%) |
| Number of bacterial STIs before dose 1, past 3 years |  |  |  |
| 0 | 26,155 | 16,285 (62.3%) | 9,870 (37.7%) |
| 1 | 2,690 | 1,597 (59.4%) | 1,093 (40.6%) |
| 2 | 1,236 | 748 (60.5%) | 488 (39.5%) |
| 3 | 677 | 403 (59.5%) | 274 (40.5%) |
| ≥4 | 2,254 | 1,359 (60.3%) | 895 (39.7%) |
| Number of bacterial STIs >3 months^b^ after dose 1 |  |  |  |
| 0 | 27,051 | 17,069 (63.1%) | 9,982 (36.9%) |
| 1 | 2,498 | 1,349 (54.0%) | 1,149 (46.0%) |
| 2 | 1,199 | 700 (58.4%) | 499 (41.6%) |
| 3 | 764 | 447 (58.5%) | 317 (41.5%) |
| ≥4 | 1,500 | 827 (55.1%) | 673 (44.9%) |
| History of HIV diagnosis before dose 1 |  |  |  |
| No | 28,421 | 17,804 (62.6%) | 10,617 (37.4%) |
| Yes | 4,591 | 2,588 (56.4%) | 2,003 (43.6%) |
| Number of physician office visits before dose 1, in past year |  |  |  |
| No visits | 5,012 | 3,344 (66.7%) | 1,668 (33.3%) |
| 1-2 visits | 6,274 | 3,964 (63.2%) | 2,310 (36.8%) |
| 3-4 visits | 5,615 | 3,400 (60.6%) | 2,215 (39.4%) |
| 5+ visits | 16,111 | 9,684 (60.1%) | 6,427 (39.9%) |
| Has a primary care physician |  |  |  |
| Not rostered | 2,641 | 1,759 (66.6%) | 882 (33.4%) |
| Rostered | 23,146 | 13,980 (60.4%) | 9,166 (39.6%) |
| Virtually Rostered | 7,225 | 4,653 (64.4%) | 2,572 (35.6%) |
| PrEP prescription before dose 1, in past year |  |  |  |
| No | 31,620 | 19,520 (61.7%) | 12,100 (38.3%) |
| Yes | 1,392 | 872 (62.6%) | 520 (37.4%) |
| PrEP prescription >3 months^a^ after dose 1 |  |  |  |
| No | 31,350 | 19,429 (62.0%) | 11,921 (38.0%) |
| Yes | 1,662 | 963 (57.9%) | 699 (42.1%) |
| Moderately or severely immunocompromised  (other than HIV) |  |  |  |
| No | 30,233 | 18,779 (62.1%) | 11,454 (37.9%) |
| Yes | 2,779 | 1,613 (58.0%) | 1,166 (42.0%) |

STI = sexually transmitted infection. PrEP = pre-exposure prophylaxis.

1. Due to institutional privacy policies, any cells ≤5 (except for missing values) must be suppressed and ranges must be provided for complementary cells to prevent back calculation
2. 3-month lag since healthcare engagement may have increased after dose 1 visit; intent was to assess potential for ongoing exposure to mpox
