# Supplementary material for: Characteristics of individuals who received a complete, 2-dose mpox vaccine regimen as part of the public health response to the mpox epidemic in Ontario, Canada
Source: PLOS Glob Public Health. 2025 Nov 26;5(11):e0005452. doi: 10.1371/journal.pgph.0005452 (PMC12654912; doi:10.1371/journal.pgph.0005452)
Supplement: S3 Table — (DOCX) [file pgph.0005452.s007.docx]

**S3 Table.** Factors associated with dose 2 receipt versus only receiving dose 1 of MVA-BN vaccine in Ontario, Canada, between June 6, 2022, to October 31, 2023, without age adjustment and with age adjustment.

| **Variable** | **Unadjusted PR (95% CI)** | **Age-adjusted PR (95% CI)** |
| --- | --- | --- |
| Sex |  |  |
| Female | Ref | Ref |
| Male | 1.16 (1.14-1.17) | 1.14 (1.12-1.16) |
| Reason for immunization |  |  |
| Pre-exposure | Ref | Ref |
| Post-exposure | 0.81 (0.78-0.84) | 0.81 (0.78-0.84) |
| Geographic region |  |  |
| Toronto | Ref | Ref |
| Rest of Ontario | 1.06 (1.04-1.07) | 1.06 (1.04-1.07) |
| Hamilton, Niagara, London | 0.97 (0.96-0.99) | 0.97 (0.96-0.99) |
| Ottawa | 1.07 (1.06-1.09) | 1.08 (1.06-1.09) |
| Peel, York, Durham, Halton | 0.93 (0.91-0.94) | 0.93 (0.92-0.95) |
| Neighbourhood income quintile |  |  |
| 1 (lowest) | Ref | Ref |
| 2 | 1.01 (1.00-1.02) | 1.01 (1.00-1.03) |
| 3 | 1.02 (1.00-1.03) | 1.02 (1.01-1.03) |
| 4 | 1.00 (0.99-1.02) | 1.01 (1.00-1.02) |
| 5 (highest) | 1.01 (1.00-1.02) | 1.01 (1.00-1.02) |
| Neighbourhood visible minorities quintile |  |  |
| 1 (lowest) | Ref | Ref |
| 2 | 0.98 (0.96-1.01) | 0.98 (0.96-1.01) |
| 3 | 0.97 (0.95-0.99) | 0.97 (0.95-0.99) |
| 4 | 0.96 (0.94-0.98) | 0.97 (0.95-0.99) |
| 5 (highest) | 0.94 (0.93-0.96) | 0.95 (0.93-0.97) |
| Recent immigration |  |  |
| Born in Canada or  immigrated before 1985 | Ref | Ref |
| Refugees | 0.93 (0.91-0.95) | 0.93 (0.91-0.95) |
| <5 years ago | 0.99 (0.98-1.01) | 1.01 (1.00-1.03) |
| 5-10 years ago | 0.99 (0.96-1.01) | 1.00 (0.98-1.02) |
| >10 years ago (but after1985) | 0.98 (0.97-1.00) | 0.98 (0.97-0.99) |
| Received any vaccine before dose 1, in past year (COVID-19, influenza, or other) |  |  |
| No | Ref | Ref |
| Yes | 1.14 (1.12-1.15) | 1.12 (1.11-1.14) |
| Number of syphilis screening tests before dose 1, in past year |  |  |
| 0 | Ref | Ref |
| 1 | 1.04 (1.03-1.05) | 1.04 (1.03-1.05) |
| 2 | 1.07 (1.06-1.08) | 1.07 (1.06-1.08) |
| 3 | 1.08 (1.07-1.10) | 1.09 (1.07-1.10) |
| ≥4 | 1.12 (1.10-1.13) | 1.12 (1.11-1.14) |
| Number of syphilis screening tests >3 months^a^ after dose 1 |  |  |
| 0 | Ref | Ref |
| 1 | 1.04 (1.03-1.05) | 1.04 (1.03-1.06) |
| 2 | 1.08 (1.07-1.10) | 1.09 (1.07-1.10) |
| 3 | 1.10 (1.09-1.12) | 1.11 (1.09-1.12) |
| ≥4 | 1.18 (1.16-1.19) | 1.19 (1.18-1.20) |
| Number of bacterial STIs before dose 1, in past 3 years |  |  |
| 0 | Ref | Ref |
| 1 | 1.02 (1.01-1.04) | 1.03 (1.02-1.05) |
| 2 | 1.01 (0.99-1.03) | 1.02 (1.00-1.04) |
| 3 | 1.02 (0.99-1.05) | 1.03 (1.00-1.06) |
| ≥4 | 1.01 (1.00-1.03) | 1.02 (1.00-1.03) |
| Number of bacterial STIs >3 months^a^ after dose 1 |  |  |
| 0 | Ref | Ref |
| 1 | 1.07 (1.05-1.08) | 1.08 (1.06-1.09) |
| 2 | 1.03 (1.01-1.06) | 1.04 (1.02-1.06) |
| 3 | 1.03 (1.01-1.06) | 1.04 (1.01-1.06) |
| ≥4 | 1.06 (1.04-1.08) | 1.07 (1.05-1.08) |
| History of HIV diagnosis |  |  |
| No | Ref | Ref |
| Yes | 1.05 (1.03-1.06) | 1.03 (1.02-1.04) |
| Number of physician office visits before dose 1, in past year |  |  |
| No visits | Ref | Ref |
| 1-2 visits | 1.03 (1.01-1.04) | 1.02 (1.01-1.03) |
| 3-4 visits | 1.05 (1.03-1.06) | 1.04 (1.02-1.05) |
| 5+ visits | 1.05 (1.04-1.06) | 1.04 (1.02-1.05) |
| Has a primary care physician |  |  |
| Not rostered | Ref | Ref |
| Rostered | 1.05 (1.03-1.06) | 1.03 (1.02-1.04) |
| Virtually Rostered | 1.02 (1.00-1.03) | 1.01 (1.00-1.03) |
| PrEP prescription before dose 1, in past year |  |  |
| No | Ref | Ref |
| Yes | 0.99 (0.97-1.01) | 1.00 (0.98-1.02) |
| PrEP prescription >3 months^a^ after dose 1 |  |  |
| No | Ref | Ref |
| Yes | 1.03 (1.01-1.05) | 1.04 (1.02-1.05) |
| Moderately or severely immunocompromised  (other than HIV) |  |  |
| No | Ref | Ref |
| Yes | 1.03 (1.02-1.04) | 1.01 (0.99-1.02) |

PR = prevalence ratio. STI = sexually transmitted infection. PrEP = pre-exposure prophylaxis. a) 3-month lag since healthcare engagement may have increased after dose 1 visit; intent was to assess potential for ongoing exposure to mpox.
