# Supplementary material for: Characteristics of individuals who received a complete, 2-dose mpox vaccine regimen as part of the public health response to the mpox epidemic in Ontario, Canada
Source: PLOS Glob Public Health. 2025 Nov 26;5(11):e0005452. doi: 10.1371/journal.pgph.0005452 (PMC12654912; doi:10.1371/journal.pgph.0005452)
Supplement: Attachment — Submitted filename: Response Letter.docx [file pgph.0005452.s009.docx]

**Response Letter**

Journal Requirements:

Please review your reference list to ensure that it is complete and correct. If you have cited papers that have been retracted, please include the rationale for doing so in the manuscript text, or remove these references and replace them with relevant current references. Any changes to the reference list should be mentioned in the rebuttal letter that accompanies your revised manuscript. If you need to cite a retracted article, indicate the article’s retracted status in the References list and also include a citation and full reference for the retraction notice.

Response: We have reviewed our reference list and confirm it is complete and correct.

Additional Editor Comments (if provided):

1. Please send a completed 'Competing Interests' statement, including any COIs declared by your co-authors. If you have no competing interests to declare, please state "The authors have declared that no competing interests exist". Otherwise please declare all competing interests beginning with the statement "I have read the journal's policy and the authors of this manuscript have the following competing interests:"

Response: We have completed the ‘Competing Interest’ statement on the submission	portal and the content is also included in the main text, Page 23, Lines 381-391.

2. Please ensure that your Ethics Statement is available in its entirety at the beginning of your Methods section, under a subheading 'Ethics Statement'.

 	Response: We have included the statement in the Methods section (Page 5, Lines 112-	117).

“***Ethics Statement***

*Ethics approval was obtained from Public Health Ontario’s Ethics Review Board. The*	*data were accessed on October 31, 2023 (the last date of the data extraction) for this*	*study. ICES is an independent, non-profit research institute whose legal status under*	*Ontario’s health information privacy law allows it to collect and analyze health care and*	*demographic data, without consent, for health system evaluation and improvement.*”

3. Please amend your detailed Financial Disclosure statement. This is published with the article. It must therefore be completed in full sentences and contain the exact wording you wish to be published.

Response: We have amended as suggested (Page 21-22, Lines 343-354).

i. State the initials, alongside each funding source, of each author to receive each grant.

 	Response: The details have been added. Page 21-22, Lines 343-354.

“*This study also received funding from the Canadian Immunization Research Network*	*through a grant from the Public Health Agency of Canada and the Canadian Institutes of*	*Health Research (CNF 151944, to JCK). The Canada-Africa Mpox Partnership, of which*	*this study is one component, was also supported by the Canadian Institutes of Health*	*Research Rapid Mpox Research (MRR-184812 to DHST)*.”

ii. State what role the funders took in the study. If the funders had no role in your study, please state: “The funders had no role in study design, data collection and analysis, decision to publish, or preparation of the manuscript.”

 	Response: Done. Page 22, Lines 351-354.

“*The funders had no role in study design, data collection and analysis, decision to*	*publish, or preparation of the manuscript. The opinions, results, and conclusions*	*reported in this paper are those of the authors and are independent from the funding*	*sources.*”

4. We have noticed that you have uploaded Supporting Information files, but you have not included a list of legends. Please add a full list of legends for your Supporting Information files after the references list.

Response: We have added a list of legends for the Supporting Information files, and	uploaded each supporting file separately as per the journal instructions. Page 20, Lines	310-322.

***“SUPPLEMENTARY FILES***

***Supplementary Methods.*** *Description of linked data repositories.*

***Figure S1.*** *Conceptual framework for associations explored between characteristics and*	*MVA-BN dose 2 receipt.*

***Figure S2.*** *Flow chart of population included in study.*

***Figure S3.*** *Time in days between first and second dose among individuals who had first*	*dose before September 30, 2022 versus on/after September 30, 2022.*

***Table S1.*** *Definitions of variables used.*

***Table S2.*** *Descriptive characteristics of individuals who received at least 1 dose of the*	*MVA-BN vaccine in Ontario, Canada, between June 6, 2022, to October 31, 2023,*	*stratified by those who received dose 2 versus those who received only dose 1, and*	*presented with row percentages.*

***Table S3****. Factors associated with dose 2 receipt versus only receiving dose 1 of MVA-*	*BN vaccine in Ontario, Canada, between June 6, 2022, to October 31, 2023, without age*	*adjustment and with age adjustment.”*

5. In the online submission form, you indicated that “The full dataset creation plan and underlying analytic code are available from the authors upon request, understanding that the computer programs may rely upon coding templates or macros that are unique to ICES and are therefore either inaccessible or may require modification.”.

All PLOS journals now require all data underlying the findings described in their manuscript to be freely available to other researchers, either

1. In a public repository,

2. Within the manuscript itself, or

3. Uploaded as supplementary information.

This policy applies to all data except where public deposition would breach compliance with the protocol approved by your research ethics board. If your data cannot be made publicly available for ethical or legal reasons (e.g., public availability would compromise patient privacy), please explain your reasons by return email and your exemption request will be escalated to the editor for approval. Your exemption request will be handled independently and will not hold up the peer review process, but will need to be resolved should your manuscript be accepted for publication. One of the Editorial team will then be in touch if there are any issues.

Response: We have updated our Data Availability statement (Page 22-23, Lines 365-380)	to provide further clarification as per the legal restrictions that prohibit us from sharing	the dataset publicly. Legal restrictions and data sharing agreements prohibit ICES from	making the dataset available. To facilitate this analysis, we transferred data from Public	Health Ontario, who are unable to disclose underlying data due to legal obligations.

*“The dataset from this study is held securely in coded form at ICES. While legal data*	*sharing agreements between ICES and data providers (e.g., healthcare organizations and*	*government) prohibit ICES from making the dataset publicly available, access may be*	*granted to those who meet pre-specified criteria for confidential access, available at*	*www.ices.on.ca/DAS (email: das@ices.on.ca). The full dataset creation plan and*	*underlying analytic code are available from the authors upon request, understanding that*	*the computer programs may rely upon coding templates or macros that are unique to*	*ICES and are therefore either inaccessible or may require modification. Further, our*	*dataset includes data from Public Health Ontario (PHO) who cannot disclose the*	*underlying data. Doing so would compromise individual privacy contrary to PHO’s*	*ethical and legal obligations. Restricted access to the data may be available under*	*conditions prescribed by the Ontario Personal Health Information Protection Act, 2004,*	*the Ontario Freedom of Information and Protection of Privacy Act, the Tri-Council*	*Policy Statement: Ethical Conduct for Research Involving Humans (TCPS 2 (2022)), and*	*PHO privacy and ethics policies. Data are available for researchers who meet PHO’s*	*criteria for access to confidential data. Information about PHO’s data access request*	*process is available on-line at* [*https://www.publichealthontario.ca/en/data-and-*](https://www.publichealthontario.ca/en/data-and-analysis/using-data/data-requests)	*analysis/using-data/data-requests.”*

Please carefully go through the comments from both reviewers. Please focus on Reviewer 1's comments on statistical analysis.

Response: We have addressed each comment and revised as suggested.

Reviewers' comments:

Reviewer's Responses to Questions

**Comments to the Author**

1. Does this manuscript meet PLOS Global Public Health’s [publication criteria](https://can01.safelinks.protection.outlook.com/?url=https%3A%2F%2Ftrack.editorialmanager.com%2FCL0%2Fhttps%3A%252F%252Fjournals.plos.org%252Fglobalpublichealth%252Fs%252Fcriteria-for-publication%2F1%2F010f0197e2819d1f-b96aa79f-7ee6-4860-a5f3-71b303450704-000000%2FwlataqEyVuYCTOrxajZisKznws7-G0HPDwfzcLGsq-U%3D218&data=05%7C02%7Csharmistha.mishra%40utoronto.ca%7C912948a284804a34289808ddc08ef9a0%7C78aac2262f034b4d9037b46d56c55210%7C0%7C0%7C638878442497595311%7CUnknown%7CTWFpbGZsb3d8eyJFbXB0eU1hcGkiOnRydWUsIlYiOiIwLjAuMDAwMCIsIlAiOiJXaW4zMiIsIkFOIjoiTWFpbCIsIldUIjoyfQ%3D%3D%7C0%7C%7C%7C&sdata=QttYzoWoiHDUzd2gF8GHY1tqIS5mamJ%2F0ZnOYGsxLuE%3D&reserved=0)? Is the manuscript technically sound, and do the data support the conclusions? The manuscript must describe methodologically and ethically rigorous research with conclusions that are appropriately drawn based on the data presented.

Reviewer #1: Yes

Reviewer #2: Yes

2. Has the statistical analysis been performed appropriately and rigorously?

Reviewer #1: No

Reviewer #2: Yes

3. Have the authors made all data underlying the findings in their manuscript fully available (please refer to the Data Availability Statement at the start of the manuscript PDF file)?

The [PLOS Data policy](https://can01.safelinks.protection.outlook.com/?url=https%3A%2F%2Ftrack.editorialmanager.com%2FCL0%2Fhttps%3A%252F%252Fjournals.plos.org%252Fglobalpublichealth%252Fs%252Fdata-availability%2F1%2F010f0197e2819d1f-b96aa79f-7ee6-4860-a5f3-71b303450704-000000%2F-KyXZPyXtyeFkluV3JJx42vaWSWcaPGZDkNrjU1RTpM%3D218&data=05%7C02%7Csharmistha.mishra%40utoronto.ca%7C912948a284804a34289808ddc08ef9a0%7C78aac2262f034b4d9037b46d56c55210%7C0%7C0%7C638878442497608788%7CUnknown%7CTWFpbGZsb3d8eyJFbXB0eU1hcGkiOnRydWUsIlYiOiIwLjAuMDAwMCIsIlAiOiJXaW4zMiIsIkFOIjoiTWFpbCIsIldUIjoyfQ%3D%3D%7C0%7C%7C%7C&sdata=Qo2h2jgjRkngahvPCgb315xJDX2C5ERvuAB7satXCw0%3D&reserved=0) requires authors to make all data underlying the findings described in their manuscript fully available without restriction, with rare exception. The data should be provided as part of the manuscript or its supporting information, or deposited to a public repository. For example, in addition to summary statistics, the data points behind means, medians and variance measures should be available. If there are restrictions on publicly sharing data—e.g. participant privacy or use of data from a third party—those must be specified.

Reviewer #1: No

Reviewer #2: No

4. Is the manuscript presented in an intelligible fashion and written in standard English?

PLOS Global Public Health does not copyedit accepted manuscripts, so the language in submitted articles must be clear, correct, and unambiguous. Any typographical or grammatical errors should be corrected at revision, so please note any specific errors here.

Reviewer #1: Yes

Reviewer #2: Yes

5. Review Comments to the Author

Please use the space provided to explain your answers to the questions above. You may also include additional comments for the author, including concerns about dual publication, research ethics, or publication ethics. (Please upload your review as an attachment if it exceeds 20,000 characters)

Reviewer #1: The study addresses a timely issue in the aftermath of the 2022 Mpox outbreak, examining factors associated with receiving the second dose of MVA-BN in Ontario. It identifies key barriers, such as access and healthcare engagement, which can inform interventions to improve vaccine coverage. However, it is necessary to address questions that remain unclear in the analysis and presentation of the results.

1. It is necessary to include a flowchart of population selection following the STROBE guide

Response: We provide a flowchart as suggested, as Supplementary Figure S2.

2. In the study design section, it is necessary to specify that this is an observational study, indicate the type of cohort, and clarify whether it is retrospective or prospective

Response: We have clarified the study is an observational, cross-sectional study using	cohort data. Methods section on study design, Page 5, Line 119.

“*We used an observational, cross-sectional study using retrospective cohort data*”

3. In the analysis, it is unclear why the authors used prevalence ratios (PR), which are primarily used in cross-sectional studies, to identify associations instead of relative risks (RR). Although the analyses were conducted using Poisson regression and the results are presented as RRs, this approach is not standard. Therefore, the authors should justify this choice.

Response: Thank you for this helpful suggestion. We have clarified that the study is a cross-sectional study (Page 5, Line 119) and thus, we report prevalence ratios.

4. In the analysis, it is necessary to first conduct normality tests for numerical variables, and then determine whether Table 1 presents the mean with ± SD or the median with IQR — but not both.

Response: Thank you, we have conducted the normality tests for the numerical variables in Table 1. We removed the means and report only the median and IQR accordingly in the revised Table 1 and provided additional information in the revised Supplementary Methods.

Supplementary Methods, Page 3:

“*For the continuous variables in Table 1 (age, days between first and second dose, rate of syphilis tests, and rate of bacterial STIs), visual inspection of the distribution of this continuous variable and tests of normality (Kolmogorov-Smirnov, Cramer-von Mises and Anderson-Darling) demonstrated a skewed distribution. Thus, the median and inter-quartile range was reported, and the Kruskal-Wallis test used for comparison across groups.*”

5. Likewise, normality analyses for numerical variables should clarify whether it is appropriate to use the Kruskal-Wallis test or the Student's t-test to compare group means.

Response: We have clarified the tests used to compare values in the footnote of Table 1 and removed the means from Table 1 as appropriate following the reporting of tests of normality (Supplementary Methods).

Table 1 footnote (Page 13, Line 204): “*Chi-squared tests were used to compare proportions. For continuous values, we used non-parametric tests of comparison (Kruskal-Wallis test)*.”

Supplementary Methods, Page 3:

“*For the continuous variables in Table 1 (age, days between first and second dose, rate of syphilis tests, and rate of bacterial STIs), visual inspection of the distribution of this continuous variable and tests of normality (Kolmogorov-Smirnov, Cramer-von Mises and Anderson-Darling) demonstrated a skewed distribution. Thus, the median and inter-quartile range was reported, and the Kruskal-Wallis test used for comparison across groups.*”

6. The use of a standardized difference ≥0.1 as 'programmatically significant' is an arbitrary threshold commonly applied in observational studies, particularly for assessing covariate balance before and after matching or weighting procedures such as propensity score matching or inverse probability weighting. However, this threshold does not necessarily imply clinical or statistical significance relevant to the study. It is unclear whether this value was used to select variables for the adjusted analysis or to assess the success of the matching or adjustment. It is important to specify its intended purpose in the results; otherwise, it would be preferable not to mention it.

Response: Thank you for this helpful suggestion. We agree and have removed the statement from the methods. We instead indicate how we looked at the confidence interval to help discern associations in the Methods, Page 7, Lines 154-163.

*“We described our sample using means and proportions and compared individuals who received only 1 dose of MVA-BN to those who received 2 doses using standardized differences, chi-square tests for categorical variables, and the Kruskal Wallis test for continuous variables. We examined the distribution of days between doses among those who received their first dose before September 30, 2022, and separately for those who received their first dose on or after September 30, 2022 (i.e., after second doses were made available). We used Poisson regression with robust standard errors adjusted for age to produce prevalence ratios (PR) with 95% confidence intervals (CI) to examine the association between the characteristics of interest and MVA-BN dose 2 receipt among dose 1 recipients. We examined the CI to discern associations.*”

7. In the analyses, why was no variable other than age included in the adjusted model, despite the epidemiological and clinical importance of other factors? I believe that, in addition to age, sex should be included as an adjustment variable, as it is associated with both the exposure (risk behaviors) and the outcome.

Response: Thank you for this suggestion. We conducted a descriptive study, focused on programmatic factors. Our study was not focused on conducting a predictive model nor to conduct causal inference. We thus focused on age-standardizing our descriptive analysis of associations. We therefore did not think it appropriate in the context of our objectives, to conduct predictive or causal modeling with further adjustment. We have done this in accordance with recommendations surrounding the conduct and importance of descriptive studies in epidemiology (Lesko CR, Fox MP, Edwards JK. A framework for descriptive epidemiology. *Am J Epidemiol*. 2022;191(12):2063–2070.)

We have provided additional rationale for this in the Methods, Page 7, Lines 163-165.

“*In accordance with our objective of a descriptive study, age-adjustment was done to age-standardize our description of associations and not to conduct predictive modeling or causal inference*.”

8. In Table 1, percentages should be calculated based on row totals rather than column totals. This approach facilitates a clearer comparison between covariates and the event of interest, and it is also the standard practice for presenting percentages.

Response: We have added a Supplementary Table (Table S2) with the row percentages.

9. In Table 2, for the variables Neighbourhood Income Quintile and Neighbourhood Visible Minorities Quintile, missing values are excluded from the analyses; this should ideally be noted in a table footnote.

Response: We have added this in the footnote of Table 2 (Page 16, Line 233)

*“^a^Missing values were excluded from analyses.”*

10. In Table 2, the first column (Received 2 doses) repeats the values from Table 1, but with a different percentage calculation. I suggest replacing this column with the results of a crude (unadjusted) analysis, which would help clarify whether any changes occurred in the adjusted analysis.

Response: We have provided a Supplementary Table S3 with the unadjusted and age-adjusted prevalence ratios and confidence intervals. Our goal was not to compare what happens due to adjustment for age, but rather to provide an age-standardized measure of association. Thus, we propose that this information (with the crude alongside the age-adjusted estimates) would be best suited as supplementary material. We note that the confidence intervals remain relatively robust to the adjustment by age (in magnitude and direction), and we highlight this in the footnote of Table S3.

Reviewer #2: Thank you for the opportunity to review your manuscript, "Characteristics of individuals who received a complete, 2-dose mpox vaccine regimen as part of the public health response to the mpox epidemic in Ontario, Canada: A CIRN study."

This is a timely and important study that addresses a critical public health issue regarding mpox vaccine series completion in Ontario. The use of a large, population-based dataset is a significant strength. I offer the following comments and suggestions to help clarify and strengthen the manuscript for publication.

Response: Thank you for the positive feedback and helpful suggestions to strengthen the	manuscript.

Major Comments
Methodological Clarity and Justification of Effect Measure: The study's design and analytical choices require further clarification.

1. Study Design: In the abstract (lines 44-49) and Methods section, the study design is not explicitly stated. While it appears to be a retrospective cohort study using secondary administrative data, this should be clearly articulated for the reader. Please specify the study design and explicitly state that this was an analysis of secondary data.

Response: We have clarified the study design and use of secondary data. Methods section on study design, Page 5, Lines 119-121):

“*We conducted an observational, cross-sectional study using retrospective cohort data. The secondary data come from linked public health and health administrative databases from the province of Ontario, Canada*.”

2. Choice of Prevalence Ratio (PR): The use of a prevalence ratio as the measure of association is a critical methodological point that needs robust justification. The study follows a cohort of individuals over a period (June 2022 to October 2023) to ascertain an outcome (receipt of a second vaccine dose). In such a longitudinal design, a risk ratio (RR) or incidence rate ratio (IRR) is typically the more appropriate measure of association, as it quantifies the probability or rate of the outcome occurring over time. A prevalence ratio is generally reserved for cross-sectional studies where exposure and outcome are measured at a single point in time. Please provide a detailed rationale for choosing the prevalence ratio over a risk ratio. If the analysis was conducted as a cross-sectional assessment at the end of the study period, this must be explicitly stated and justified as the optimal approach for your research question. Without this justification, the strength and interpretation of the findings are unclear.

Response: We have clarified the cross-sectional nature of the study design. That is, we do not use a cohort study design, and we acknowledge having created some confusion in the original version of the manuscript by using that term. Because we used a cross-sectional study design, we therefore used prevalence ratios to describe associations as noted by the reviewer above.

Minor Comments

Title: The title is informative but contains an abbreviation ("CIRN") that may not be familiar to all readers. Per journal guidelines, it is best practice to avoid abbreviations in the title. Please spell out the Canadian Immunization Research Network or remove the acronym.

Response: We have removed the acronym from the title.

Abstract:

Methods (Lines 44-49): The description of the methods is vague. Please clarify the study population and how individuals were identified and included. Stating that you used "provincial health administrative data" is good, but a more explicit description of the cohort definition would be beneficial.

Response: We edited the Abstract to provide a more explicit description including study design, and provide a more detailed explanation in the Methods section of the main text.

Abstract (Page 2, Lines 45-46):

*“We conducted a cross-sectional study using used population-level data among individuals who received ≥1 dose of MVA-BN between June 6, 2022 and October 31, 2023 in Ontario…”*

Methods (Page 5-6, Lines 119-126).

“*We conducted an observational, cross-sectional study using retrospective cohort data. The secondary data come from linked public health and health administrative databases from the province of Ontario, Canada. The province has a single payer healthcare system, which means that there is a single repository for health data on all individuals who are residents of Ontario and who receive health care through the single payer system. Thus, the health administrative databases include person-level data on vaccination, laboratory tests, as well as neighborhood-level measures. The details of the health administrative data sources are provided in the Supplementary Material: Supplementary Methods.”*

Results (Line 52): The phrasing "e.g., Ottawa versus Toronto" is ambiguous. Does this mean this is just one example of a comparison, or is this the specific comparison being reported? Please rephrase for clarity, for instance: "higher in Ottawa compared to Toronto..."

Response: Edited to improve precision and clarity as suggested.

Abstract (Page 2, Lines 52-53):

*” Receipt of dose 2 versus only dose 1 was associated with region (e.g., higher in Ottawa compared with Toronto…”*

Conclusion (Lines 58-59): The objective was to understand how individuals who received two doses differed from those who received only one. The conclusion should more directly summarize these key differences that were identified. For example, "Individuals who completed the 2-dose series were more likely to have higher healthcare engagement, as evidenced by..."

Response: Thank you for this suggestion. However, we propose that conclusions would best provide a broad summary rather than repeat the findings from the results and provide a “so what” statement re: potential implications of the findings. We also recognize the limitation on word count for the abstract. We hope this explains our rationale for the current content of the conclusion statement in the abstract.

Introduction:

Line 103: The statement regarding a sustained increase in mpox cases in 2024 is a strong justification for the study's importance. Please provide a citation to support this claim.

Response: We have added a citation which describes the epidemiology of mpox in Ontario in 2024 to Page 5, Line 104 as citation number 7:

Ontario Agency for Health Protection and Promotion (Public Health Ontario). Enhanced epidemiological summary: Mpox in Ontario: January 1, 2024 to December 31, 2024. Toronto, ON: King’s Printer for Ontario; 2025.

General:

Please ensure consistency in the style of in-text citations throughout the manuscript to conform to the journal's formatting requirements.

Response: We have checked and corrected to the journal’s specifications.

I trust these comments will be useful in revising your manuscript. I look forward to seeing a revised version that addresses these points.

Response: Thank you for the very helpful suggestions.

6. PLOS authors have the option to publish the peer review history of their article ([what does this mean?](https://can01.safelinks.protection.outlook.com/?url=https%3A%2F%2Ftrack.editorialmanager.com%2FCL0%2Fhttps%3A%252F%252Fjournals.plos.org%252Fglobalpublichealth%252Fs%252Feditorial-and-peer-review-process%2523loc-peer-review-history%2F1%2F010f0197e2819d1f-b96aa79f-7ee6-4860-a5f3-71b303450704-000000%2FMBSPxH_ILVy_D7YJIOhpraDvXukKY-Y958BqO5reSss%3D218&data=05%7C02%7Csharmistha.mishra%40utoronto.ca%7C912948a284804a34289808ddc08ef9a0%7C78aac2262f034b4d9037b46d56c55210%7C0%7C0%7C638878442497621126%7CUnknown%7CTWFpbGZsb3d8eyJFbXB0eU1hcGkiOnRydWUsIlYiOiIwLjAuMDAwMCIsIlAiOiJXaW4zMiIsIkFOIjoiTWFpbCIsIldUIjoyfQ%3D%3D%7C0%7C%7C%7C&sdata=t2Kt1Wwtl3tN%2BRm2KH3VofWz2IZj0XSGa10g%2BkWtCr4%3D&reserved=0)). If published, this will include your full peer review and any attached files.


**Do you want your identity to be public for this peer review?** If you choose “no”, your identity will remain anonymous but your review may still be made public.


For information about this choice, including consent withdrawal, please see our [Privacy Policy](https://can01.safelinks.protection.outlook.com/?url=https%3A%2F%2Ftrack.editorialmanager.com%2FCL0%2Fhttps%3A%252F%252Fwww.plos.org%252Fprivacy-policy%2F1%2F010f0197e2819d1f-b96aa79f-7ee6-4860-a5f3-71b303450704-000000%2F0-AmhPfWr2TtxCN-OMe1W9cT3BlxTxkNBJJoXMbOK4A%3D218&data=05%7C02%7Csharmistha.mishra%40utoronto.ca%7C912948a284804a34289808ddc08ef9a0%7C78aac2262f034b4d9037b46d56c55210%7C0%7C0%7C638878442497637614%7CUnknown%7CTWFpbGZsb3d8eyJFbXB0eU1hcGkiOnRydWUsIlYiOiIwLjAuMDAwMCIsIlAiOiJXaW4zMiIsIkFOIjoiTWFpbCIsIldUIjoyfQ%3D%3D%7C0%7C%7C%7C&sdata=iJLfmRzSA9Zn5PZ%2FrCeyKYryseYXoLCOFh4s9%2BsVK9E%3D&reserved=0).

Reviewer #1: **Yes:** Hugo Arroyo-Hernández

Reviewer #2: **Yes:** Suleiman Idris Ahmad
